# Molecularly Imprinted Membranes: Dual@MIPs@mbr for On-Site Detection of CA 19-9

**DOI:** 10.3390/s25237363

**Published:** 2025-12-03

**Authors:** Eduarda Rodrigues, Ana Xu, Paula Sampaio, Rafael C. Castro, David S. M. Ribeiro, João L. M. Santos, Ana Margarida L. Piloto

**Affiliations:** 1 CIETI-LabRISE, ISEP, Polytechnic of Porto, Rua Dr. António Bernardino de Almeida 431, 4249-015 Porto, Portugal; edmfr@isep.ipp.pt (E.R.); naaxu@isep.ipp.pt (A.X.); 2 i3S—Institute of Research and Innovation in Health, University of Porto, 4200-135 Porto, Portugal; sampaio@i3s.up.pt; 3 IBMC—Institute for Molecular and Cell Biology, University of Porto, 4150-180 Porto, Portugal; 4 LAQV, REQUIMTE, Laboratory of Applied Chemistry, Department of Chemical Sciences, Faculty of Pharmacy, University of Porto, Rua de Jorge Viterbo Ferreira No 228, 4050-313 Porto, Portugal; rafael.castro.cl@hotmail.com (R.C.C.); dsmribeiro@gmail.com (D.S.M.R.); joaolms@ff.up.pt (J.L.M.S.)

**Keywords:** dual-emission molecularly imprinted membranes (dual@MIPs@mbr), quantum dots (QDs), carbohydrate antigen CA 19-9, point-of-care detection

## Abstract

**Highlights:**

**What are the main findings?**

- Dual-emission imprinted membranes (dual@MIPs@mbr) were developed as a proof-of-concept ratiometric fluorescence platform for CA 19-9 detection.
- Embedding y-QDs and b-CDs enabled a visible color change from yellow/green to blue upon antigen binding.

**What are the implications of the main findings?**

- The membranes showed a linear range of 4–400 U mL^−1^ and a detection limit of 0.056 U mL^−1^ in diluted serum, with good selectivity and short-term stability.
- The method is presented as a preliminary analytical approach and requires validation in real clinical samples before further application in point-of-care biosensing.

**Abstract:**

Dual-emission molecularly imprinted membranes (dual@MIPs@mbr) were developed as a proof-of-concept platform for the selective and instrument-free detection of the cancer biomarker carbohydrate antigen 19-9 (CA 19-9). The system integrates a ratiometric fluorescence response by embedding yellow-emitting quantum dots (y-QDs), serving as target-responsive probes, and blue-emitting carbon dots (b-CDs), acting as an internal reference, within a CA 19-9-imprinted polymeric matrix. Specific rebinding of CA 19-9 to the imprinted cavities induced selective quenching of the y-QDs while preserving the b-CDs emission, yielding a visible color shift from yellow/green to blue. This behavior enabled the quantification of CA 19-9 over a linear range of 4–400 U mL^−1^, with a limit of detection of 0.056 U mL^−1^ in diluted serum. The membranes showed good selectivity against common serum interferents and maintained short-term photochemical stability. Although the method has not yet been validated using real clinical samples, the pronounced ratiometric response and simple visual readout demonstrate its potential as a low-cost, portable sensing approach for future point-of-care cancer biomarker analysis.

## 1. Introduction

Pancreatic cancer remains one of the deadliest malignancies worldwide, with a 5-year survival rate of less than 1% according to the Global Cancer Observatory [1,2]. Early diagnosis is crucial for improving patient outcomes, and the identification of reliable biomarkers for early detection and disease monitoring is a major focus of cancer diagnostics research [3,4,5]. Among the currently available biomarkers, carbohydrate antigen 19-9 (CA 19-9) is one of the most widely used indicators for pancreatic cancer. It is a high-molecular-weight glycoprotein (≈210 kDa), and elevated serum levels above 37 U mL^−1^ are commonly associated with tumor progression and poorer clinical outcome. For this reason, it is routinely employed in clinical practice for prognosis, treatment monitoring, and as a complementary marker in diagnostic panels [6]. Clinically, CA 19-9 concentrations between 100 and 500 U mL^−1^ are often observed in benign inflammatory conditions such as pancreatitis or cholestasis, whereas values exceeding 1000 U mL^−1^ are frequently linked to advanced pancreatic malignancies. These clinically established thresholds justify the analytical range selected in this work (4–400 U mL^−1^), which covers concentrations relevant for early disease detection and routine patient monitoring [7]. Several analytical methods have been developed for CA 19-9 detection, including enzyme-linked immunosorbent assays (ELISA), electrochemical and photoelectrochemical biosensors [8,9,10], fluorescence-based assays [9], surface-enhanced Raman scattering (SERS) [10], and mass spectrometry, among others [11]. However, most of these approaches involve complex instrumentation, lengthy preparation steps, and high operational costs, which limit their use in point-of-care testing. To overcome these challenges, optical biosensing—particularly colorimetric and fluorescence-based detection—has emerged as a promising alternative due to its simplicity, rapid response, and potential for visual readouts. Optical biosensors are especially advantageous in cancer diagnostics because of their compatibility with multiplexed assays and ease of miniaturization [12].

Molecularly imprinted polymers (MIPs) are synthetic materials that emulate the molecular recognition properties of biological receptors such as antibodies and enzymes [13]. Their three-dimensional structure contains specific binding sites that are complementary in shape, size, and functional groups to the target molecule, enabling selective recognition [14]. MIPs can be fabricated via various imprinting strategies, including bulk, surface, epitope, and nanoimprinting approaches [15]. In surface imprinting, for instance, the target molecule is first complexed with functional monomers and cross-linkers to form a pre-polymerization assembly; subsequent polymerization and removal of the target leave behind “molecular memory” cavities capable of rebinding the analyte with high specificity [14,16].

Quantum dots (QDs) are excellent fluorescent probes for biosensing due to their high brightness, chemical stability, and tunable optical properties, which make them particularly suitable for cancer detection and imaging [17,18]. However, single-emission-based fluorescence assays are prone to background interference and instrumental variability. To address these limitations, ratiometric fluorescence approaches have been developed, in which two distinct emission signals are used—one responsive to the analyte and another serving as an internal reference [19,20]. Carbon dots (CDs) are often employed as the reference fluorophore due to their chemical inertness, low toxicity, high biocompatibility, water solubility, and strong fluorescence [21]. CDs have been widely applied in bioimaging, chemical sensing, and diagnostic platforms [18,19,22,23].

Paper-based and membrane-based analytical devices have also attracted considerable attention for their portability, low cost, and potential for use in resource-limited settings [24]. These solid supports—such as silica gel plates, acid-free papers, or porous polymer membranes—facilitate reagent immobilization and signal visualization [25]. MIP-based paper or membrane sensors combine the selectivity of imprinting with the convenience of disposable analytical devices, representing a powerful alternative for biomarker detection and clinical screening [26,27].

In this study, dual-emission molecularly imprinted membranes (dual@MIPs@mbr) were designed and fabricated for the selective and instrument-free detection of CA 19-9. The innovation of this work lies in the integration of yellow-emitting quantum dots (y-QDs) as target-responsive probes and blue-emitting carbon dots (b-CDs) as internal fluorescence references within a polymeric matrix, as dual-emission molecularly imprinted polymers (dual@MIPs). This dual emission system enables a ratiometric optical response (I_blue_/I_yellow_) that allows visual and quantitative detection without the need for external calibration. The goal of this work was to establish a preliminary, low-cost sensing strategy with selective recognition, reproducibility, and short-term photostability suitable for future point-of-care CA 19-9 monitoring. To this end, CA 19-9-imprinted membranes were synthesized using a surface imprinting method, in which the antigen acted as a template during polymerization and was subsequently removed to create selective binding cavities. The resulting membranes were systematically characterized and evaluated for their optical behavior, analytical performance, and selectivity against common serum interferents, laying the groundwork for future validation in real clinical samples.

## 2. Materials and Methods

### 2.1. Chemicals and Samples

Tellurium powder (200 mesh, 99.8%) was obtained from Alfa Aesar (Quimigen, Alverca do Ribatejo, Portugal); sodium borohydride, ammonium persulfate (APS) and tetramethyl ethylenediamine (TEMED) were purchased from Fisher Chemical (Quimigen, Alverca do Ribatejo, Portugal); 3-mercaptopropionic acid (MPA, 99%), 2-aminoethyl methacrylate hydrochloride (AEMH·HCl), acrylamide (AAM) and N, N′-methylenebis(acrylamide) (MBA) were obtained from Sigma-Aldrich (VWR International, Amadora, Portugal); cadmium chloride hemi(pentahydrate) (CdCl_2_·2.5H_2_O) 98% and citric acid were acquired from Acros Organics (Fisher Scientific, Porto Salvo, Portugal); sodium hydrogen carbonate (NaHCO_3_) and ethylenediamine (C_2_H_8_N_2_) were purchased from Panreac Quimica SA (Quimigen, Alverca do Ribatejo, Portugal); sodium carbonate decahydrate (Na_2_CO_3_·10H_2_O)was acquired from Riedel-de Haën (Fisher Scientific, Porto Salvo, Portugal); ethanol (96%) was obtained from LabChem (Labor Spirit Lda., Loures, Portugal); human CA 19-9 antigen grade protein unconjugated, was purchased from Biorbyt (Biorbyt Ltd., Cambridge, UK); lyophilized human normal (HN) serum was purchased from PZ CORMAY (PZ CORMAY S.A, Motycz, Poland); creatinine (CREA) was purchased from Fluka BioChemika (José Manuel Gomes Santos, Lda (JMGS), Porto, Portugal); mucin 4 (MUC 4) was obtained from Abbexa (Abbexa Ltd., Cambridge, UK); albumin human serum (HSA) and phosphate saline buffer (PBS) were obtained from Sigma-Aldrich Aldrich (Sigma Aldrich Química, S.A., Sintra, Portugal); polyamide membrane filters 47 mm in diameter and 0.45 µm in pore size were bought from Filter-Lab (Filtros Anoia, S.A., Barcelona, Spain). All fluorescence measurements were performed with a QS precision cell with a 1 mm light path made of quartz suprasil^®^ from Hëllma^®^Analytics (Fisher Scientific, Unipessoal, Lda, Oeiras, Portugal). All solutions were prepared with water from a Milli-Q system (specific conductivity < 0.1 µS cm^−1^) and chemicals of analytical reagent grade quality were used. Details on the instrumentation and optical analysis used throughout the experimental section are provided in the Supplementary Materials (SM) file.

### 2.2. Apparatus

Several techniques were employed for the characterization of materials, namely ultraviolet-visible spectroscopy (UV-vis), Fourier transform infrared spectroscopy (FT-IR), fluorescence spectroscopy, scanning electron microscopy (SEM), energy-dispersive X-ray spectroscopy (EDS), and fluorescence confocal microscopy.

UV-vis spectra were recorded using an Evolution 220 UV-vis spectrophotometer (Thermo Fisher Scientific Inc., Waltham, MA, USA) in the wavelength range of 200–600 nm. FT-IR spectra were acquired on a Nicolet iS10 spectrometer (Thermo Fisher Scientific Inc., Waltham, MA, USA), equipped with an attenuated total reflectance (ATR) accessory featuring a diamond contact crystal. The measurements were conducted with a resolution of 32 cm^−1^ and covered a spectral range from 3720 to 850 cm^−1^. SEM and EDS analyses were performed on a Phenom ProX Desktop SEM (Thermo Fisher Scientific, Waltham, MA, USA) operated at an accelerating voltage of 10 kV, allowing for both morphological and elemental analysis. Steady-state fluorescence spectra were collected using a Lumina fluorescence spectrometer (Thermo Scientific, Waltham, MA, USA) equipped with a 150 W continuous-wave xenon-arc discharge lamp as the excitation source. Measurements were carried out at a scanning rate of 600 nm/min, with an integration time of 50 ms and a response time of 0.02 s. The photomultiplier tube (PMT) voltage was set at either 330 or 400 V, depending on the experimental conditions, with both the excitation and emission slits set to 20 nm. Fluorescence data were collected using a Hellma Suprasil quartz cell with a 1 mm optical path (Hellma Analytics, Müllheim, Germany), with excitation performed at 390 nm and emission spectra recorded in the range of 420–720 nm. The filters were set to air for all fluorescence measurements. Fluorescence confocal microscopy images of dual@MIPs@mbr were obtained following a standardized procedure. Polyamide membranes were mounted on a glass lamellae with a coverslip for imaging (MatTek Inc., Ashland, MA, USA). The analysis was carried out using a Leica Stellaris 8 Laser Scanning Confocal Microscope (Leica Microsystems GmbH, Wetzlar, Germany). To optimize the detection of y-QDs and b-CDs fluorescence, excitation was performed at two wavelengths: 390 nm and 488 nm, with fluorescence emission detected in the range of 430–750 nm using a HyD X detector. A HC PL APO CS2 10×/0.40 objective was employed for image acquisition. Images were captured with a pixel size of 3 µm for standard images and 390 nm for zoomed-in views. All microscopy data were processed using the open-source software Fiji/ImageJ version 1.54f (National Institutes of Health, Bethesda, MD, USA), applying consistent histogram adjustments across all samples to ensure comparability across experimental conditions.

### 2.3. Synthesis of Colloidal Precursors

#### 2.3.1. y-QDs

y-QDs capped with MPA were synthesized with modifications based on Zou et al. [28,29]. In a three-necked flask, CdCl_2_·2.5H_2_O (1207 mg) and MPA (888 µL) were added to 60 mL ultrapure water, and the pH was adjusted to 11.5 using 1 M NaOH. The solution was degassed with N_2_ and refluxed under continuous stirring. In parallel, NaHTe precursor was prepared by mixing NaBH_4_ (360 mg) with Te powder (76 mg) in 5 mL ultrapure water previously degassed with N_2_. The mixture was heated to 80 °C until a deep purple color developed and was then rapidly injected into the refluxing Cd-MPA solution. The molar ratio Cd^2+^/Te^2−^/MPA was maintained at 1:0.1:1.5. After reaction completion, the suspension was precipitated with ethanol and centrifuged (8000 rpm, 5 min, 22 °C). The resulting solid was dried in a desiccator and stored protected from light at room temperature.

#### 2.3.2. b-CDs

b-CDs were synthesized following Castro et al. [30,31]. Citric acid (500 mg) and ethylenediamine (300 µL) were added to 5 mL ultrapure water, and the pH was adjusted to 3.8 with 1 M HCl. The solution was transferred to a 40 mL Teflon-lined autoclave and heated at 260 °C for 5 h. After cooling to room temperature, the suspension was dialyzed against Milli-Q water using a Spectra/Por 6 dialysis membrane (1000 MWCO) for 3 days. Purified suspensions were divided for short-term storage at −4 °C and long-term storage after lyophilization. Lyophilization was performed by freezing at −55 °C for 6 h, followed by primary drying at −45 °C under 55 mbar for 12 h and secondary drying at +20 °C under 30 mbar for 6 h. The resulting light-brown b-CDs powder was stored in amber vials at room temperature, protected from light and humidity.

Both y-QDs and b-CDs employed in this work have been previously characterized and reported regarding its hydrodynamic size, zeta potential, fluorescence quantum yield, and lifetime [29]. The same batch of nanoparticles was used herein, ensuring consistent optical and colloidal properties across studies.

#### 2.3.3. Dual@nanoparticles

Dual@nanoparticles were prepared by mixing y-QDs (2 mg), b-CDs (0.2 mg), and 200 µL of 1% HN in PBS. The mixture was incubated for 20 min at 22 °C to ensure homogeneous dispersion of both fluorophores. The resulting colloidal suspension was used for comparative fluorescence studies.

#### 2.3.4. MIP@QDs

**Figure 1:**
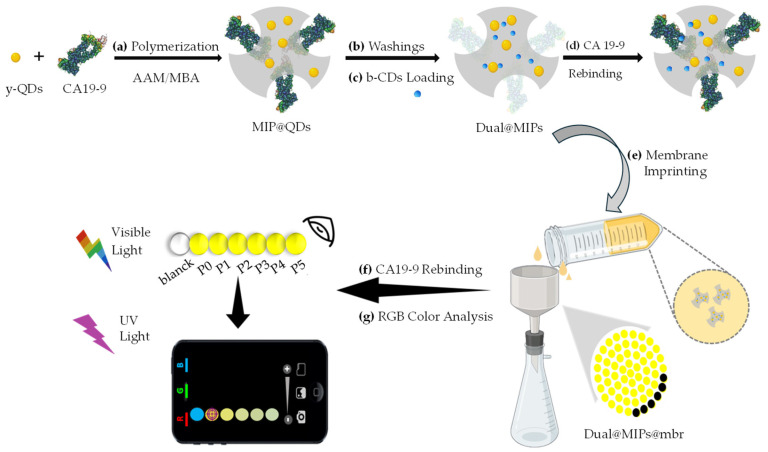
Assembly of the imprinted membranes (dual@MIPs@mbr). (**a**) Polymerization of MIP@QDs; (**b**) Washings of MIP@QDs; (**c**) Loading of b-CDs onto MIP@QDs to form dual@MIPs; (**d**) Rebinding of CA 19-9 in serum; (**e**) Imprinting of dual@MIPs onto the membranes (dual@MIPs@mbr); (**f**) CA 19-9 rebinding on the imprinted membranes; (**g**) RGB color analysis of the imprinted membranes under 365 nm UV light.

Molecularly imprinted polymer-coated quantum dots (MIP@QDs) were prepared by a surface imprinting approach adapted from Ref. [32]. Briefly, CA 19-9 (1 kU mL^−1^) was incubated for 3 h at 22 °C with y-QDs (2 mg) in PBS 10 mM (1 mL), previously adjusted to pH 6.4 using phosphoric acid. Next, AEMH·HCl (2.42 × 10^−5^ M) was added and the mixture was stirred for 30 min at 22 °C. After centrifugation (8000 rpm, 22 °C), the pellet was resuspended in PBS (1 mL) with AAM (1 × 10^−4^ M), MBA (1 × 10^−4^ M), APS (2 × 10^−3^ M) and TEMED (1 × 10^−3^ M) (Figure 1a). The polymerization mixture was degassed with N_2_ (30 min, 22 °C), followed by centrifugation (8000 rpm, 22 °C) and resuspension in PBS (1 mL). The mixture was resuspended in carbonate/bicarbonate buffer (pH 9.8) (48 h, 22 °C) under gentle agitation and centrifuged (4000 rpm, 22 °C) (Figure 1b). The resulting pellet was resuspended in PBS (1 mL) and the cycle was repeated until no template was detected in the supernatants by UV-Vis (Figure S1f).

The washed MIP@QDs were stored in PBS at 4 °C for short-term use (up to 24 h) or lyophilized for long-term storage. For lyophilization, the MIP@QDs were dialyzed against Milli-Q water (5 days, 1000 MWCO membrane), frozen at −80 °C for ≥12 h, and freeze-dried under controlled conditions. The resulting MIP@QDs powder was stored in amber glass vials, 22 °C. Non-imprinted polymer-coated quantum dots (NIP@QDs), were synthesized in parallel under identical conditions but without the addition of CA 19-9.

#### 2.3.5. Dual@MIPs

Dual@MIPs were obtained by mixing MIP@QDs (2 mg), b-CDs (0.2 mg), and 200 µL of PBS 10 mM, pH 6.4. The mixture was incubated for 20 min at 22 °C to allow stable integration of the two fluorescent components. An analogous procedure was used to prepare non-imprinted controls, dual@NIPs (Figure 1c).

### 2.4. Assembly of Dual@MIPs@mbr

Polyamide membranes (47 mm diameter, 0.45 µm pore size) were pre-hydrated with PBS before coating. Then, 5 mL of a freshly prepared suspension of dual@MIPs in PBS was filtered through the membranes under gentle vacuum (980 mbar), using a Büchner funnel, to obtain the imprinted membranes as dual@MIPs@mbr. For long-term storage, the imprinted membranes were kept at 4 °C up to 20 days, protected from light (Figure 1e). For ready-to-use experiments, the imprinted membranes were punched into 3 mm circular disks and dried at 37 °C for 1 h. The same protocol was applied to prepare non-imprinted membranes as controls, dual@NIPs@mbr.

### 2.5. Calibration Protocols

Calibrations on the colloidal precursors dual@MIPs were performed as follows. Briefly, 2 mg of MIP@QDs and 0.2 mg of b-CDs were mixed with 190 µL of 1% HN serum in PBS (Figure 1c). The suspension was then incubated with CA 19-9 standard solutions ranging from 3.96 × 10^−5^ to 5.20 × 10^−2^ U mL^−1^ for 20 min at 22 °C, with the final volume adjusted to 200 µL 1% HN in PBS. Following incubation, the suspensions were centrifuged at 8000 rpm for 5 min at 22 °C, and the supernatants were discarded. The resulting pellets were resuspended in 200 µL 1% HN in PBS. Identical procedures were applied to non-imprinted polymer controls (NIP@QDs) to prepare the dual@NIPs, and to dual@nanoparticles suspensions, to serve as comparative standards (Figure 1d).

Following protocol 2.4 for preparation of imprinted membranes, 3 mm circular disks were punched and dried at 37 °C for 1 h. Then 3 µL of CA 19-9 standard solutions in the concentration range from 0.4 to 400 U mL^−1^ in 1% HN in PBS, were incubated on the disks for 20 min at 22 °C. Then the disks were washed with PBS (100 μL) and dried at 37 °C for 1 h. Fluorescence imaging of the disks was conducted under controlled lighting conditions using a 365 nm UV lamp (UVGL-58, 5 W, 230 V, 50–60 Hz) placed inside a custom-built black box to eliminate ambient light (Figure 1f). Images were acquired using an iPhone 12 Pro positioned 18 cm above the membrane surface, configured with 12 MP resolution, f/1.6 aperture, autofocus enabled, and flash disabled. For quantification, image analysis focused on the central region of each fluorescent spot, from which the mean intensity values of the blue and green color channels were extracted (S/N = 5) (Figure 1g). The ratiometric fluorescence response (I_blue_/I_green_) was plotted against CA 19-9 concentration and analyzed using the Stern-Volmer Equation 1. This protocol was repeated with the controls dual@NIPs@mbr. Calibration data are provided in Table S3.

### 2.6. Confocal Microscopy Protocols

Confocal fluorescence microscopy was performed on imprinted membranes and its controls mounted on glass-bottom slides. Excitation wavelengths of 390 nm and 488 nm were used to visualize b-CDs and y-QDs fluorescence, respectively. Emission spectra (430–750 nm) were recorded using a HyD X detector with a HC PL APO CS2 10×/0.40 objective. Overview and high-resolution images were acquired at pixel resolutions of 3 µm and 379 nm, respectively. Image processing and analysis were conducted in Fiji/ImageJ using uniform histogram adjustments for direct comparison.

### 2.7. Selectivity Tests

Following protocol 2.4 for preparation of imprinted membranes, 3 mm circular disks were punched and dried at 37 °C for 1 h. At this time, the disks were incubated with 3 µL CA 19-9 solution (40 U mL^−1^) alone or in the presence of biological interferents—creatinine (6 µg mL^−1^), human serum albumin (4 mg mL^−1^), and mucin 4 (1.4 ng mL^−1^). Incubations were carried out for 15 min at 37 °C, followed by washing and drying under the same conditions. Each condition was tested in six replicates. The same protocol was applied to dual@NIPs@mbr as controls.

### 2.8. Stability and Reproducibility Tests

Following protocol 2.4 for preparation of imprinted membranes, 3 mm circular disks were punched and dried at 37 °C for 1 h. Reproducibility was assessed by incubating the disks with CA 19-9 standard solutions at three concentrations: 3.88 × 10^−1^, 9.62 × 10^1^, and 3.81 × 10^2^ U mL^−1^, prepared in 1% HN in PBS, in accordance with the imaging conditions described in Section 2.5.

## 3. Results and Discussion

### 3.1. Characterization of Colloidal Precursors

y-QDs and b-CDs have been synthesized and characterized in previous works from the authors [29,31]. Colloidal MIP@QDs were synthesized via a surface imprinting approach using CA 19-9 protein as the template, thereby preventing complete entrapment of the target within the polymer matrix. Following the methodology of Piloto et al. the imprinting concentration of CA 19-9 was fixed at 1 kU mL^−1^ being optimized the initial y-QDs concentration, polymerization time, TEMED concentration, and washing duration for template removal [33] (Figure S1, Table S1). Since CA 19-9 has an isoelectric point in the alkaline range (pH > 7), electrostatic interactions between the positively charged regions of the glycoprotein and the negatively charged carboxylate groups on the y-QDs surface were favored in PBS at pH 6.4 [34]. Template removal was conducted by incubating the polymerized MIP@QDs for 48 h in carbonate/bicarbonate buffer (10 mM, pH 9.8) (Figure 1b, Figure S1d blue solid line). Table S1 shows the experimental conditions used for template removal. After this treatment, the washed MIP@QDs exhibited a marked increase in fluorescence intensity relative to the as-polymerized samples, confirming efficient template removal and restoration of the y-QDs emission (Figure S1d, pink dashed line). yellow solid line In contrast, the corresponding washed NIP@QDs maintained similar fluorescence to their polymerized state, as expected due to the absence of specific template–polymer interactions (Figure S1d).

Below are the techniques employed to characterize the synthesized precursors. X-ray photoelectron spectroscopy (XPS) was not conducted because both the polyamide-based polymeric membranes and carbon-dot composites are non-conductive and beam-sensitive. Under high-vacuum and X-ray irradiation, these materials are susceptible to surface charging, structural deformation, and fluorescence degradation, which can compromise the accuracy of elemental and chemical-state analyses [35]. Consequently, low-voltage SEM and optical spectroscopy were selected as non-destructive alternatives to preserve the native morphology and photoluminescent properties of the materials.

### 3.2. FT-IR

**Figure 2:**
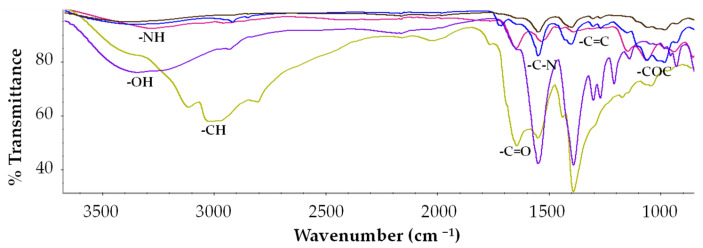
FT-IR spectra of raw y-QDs (purple line); raw b-CDs (green line); washed dual@MIPs (blue line); washed dual@NIPs (brown line) and CA 19-9 antigen (pink line).

Prior to analysis, all samples were dried in a desiccator at room temperature to remove residual moisture. The FT-IR spectra of the materials displayed distinct vibrational features characteristic of the functional groups associated with the carbon nanostructures, the CA 19-9 protein, and the polymeric matrices (Figure 2). Broad absorption bands between 3340 and 3390 cm^−1^ correspond to O–H stretching vibrations (Figure 2, purple, green, brown, and blue lines), while the band at 3285 cm^−1^ is attributed to N–H stretching, typical of peptide linkages in the CA 19-9 protein (Figure 2, pink line) [36].

The FT-IR spectrum of CA 19-9 exhibited the characteristic amide I and amide II bands at 1650 cm^−1^ and 1540 cm^−1^, respectively, corresponding to C=O stretching and C–N bending vibrations, along with a feature at 1150 cm^−1^ associated with coupled N–H and C–N stretching modes (Figure 2, pink line). Both dual@MIPs and their non-imprinted counterparts showed peaks at 1400 cm^−1^ and 980 cm^−1^, assigned to C=C stretching from unsaturated vinyl groups and C–O–C stretching of the polymeric backbone, respectively (Figure 2, blue and brown lines). The spectrum of y-QDs revealed a distinct band at 1552 cm^−1^, corresponding to the asymmetric stretching of carboxylate (COO^−^) groups (Figure 2, purple line), whereas b-CDs displayed a C=O stretching band at 1646 cm^−1^, indicative of carbonyl functionalities formed during synthesis (Figure 2, green line). Notably, dual@MIPs exhibited additional absorption bands at 1641 cm^−1^ and 1563 cm^−1^, corresponding to amide I and amide II vibrations, respectively (Figure 2, blue line) [37]. These bands, attributed to residual protein fragments retained within the imprinted cavities, were absent in the non-imprinted controls (Figure 2, brown line). The emergence of these distinctive amide bands confirms the successful molecular imprinting of the CA 19-9 template and the creation of complementary recognition sites within the polymeric matrix [18,33].

### 3.3. SEM and EDS

**Figure 3:**
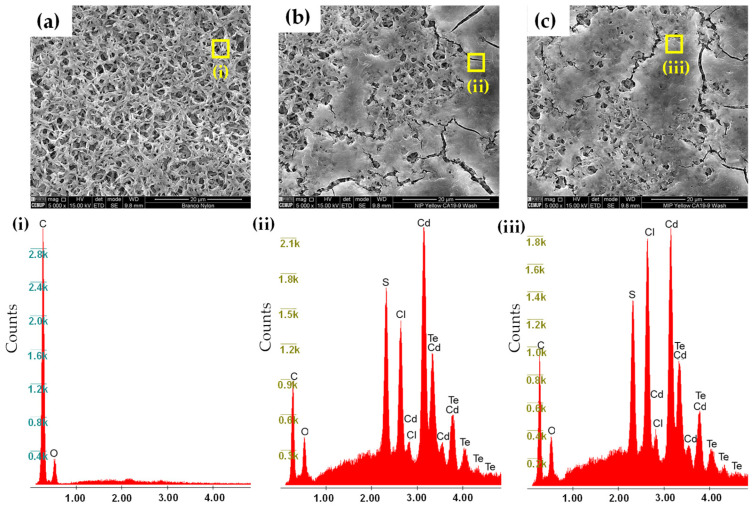
SEM images of raw polyamide membranes (**a**), non-imprinted membranes washed (**b**), and imprinted membranes washed (**c**). Correspondent spots of the EDS analysis on raw polyamide membranes (**i**), dual@NIPs@mbr (**ii**), and dual@MIPs@mbr (**iii**).

The imprinted membranes exhibited a porous surface morphology with irregular depressions and cavity-like features (Figure 3c), whereas the non-imprinted counterparts presented a more compact and homogeneous surface without visible cavities (Figure 3b). The raw polyamide substrate showed the characteristic fibrous structure of the unmodified membrane (Figure 3a). These morphological differences are consistent with polymer growth around the CA 19-9 template molecules and the subsequent formation of recognition sites. CA 19-9 is a high-molecular-weight glycoprotein (~210 kDa) whose expected hydrodynamic size lies in the low-nanometer range (≈5–15 nm) [38]. Therefore, the micron-scale depressions observed by SEM are not direct replicas of individual protein molecules. Instead, such larger features arise from (i) aggregation or multi-molecule adsorption of the protein during imprinting, (ii) polymer network relaxation and shrinkage during drying, and (iii) the intrinsic resolution limits of low-voltage SEM, which do not resolve nanoscale imprint cavities. Similar mesoscale cavity enlargement is widely reported for surface-imprinted polymers on soft polymeric substrates.

EDS analysis detected carbon, oxygen, cadmium, and tellurium on all membranes. Imprinted membranes presented slightly higher levels of carbon, sodium, and chlorine, which may reflect residual protein fragments or functional groups retained after template removal, whereas sulfur signals in both MIPs and NIPs originate from MPA-capped nanoprobes. These compositional differences further support the structural changes induced by the imprinting process. Because the polyamide support and the polymeric MIP coating are beam-sensitive, high-resolution SEM or TEM could not be performed without compromising the structural and photoluminescent integrity of the materials.

### 3.4. Confocal Microscopy

**Figure 4:**
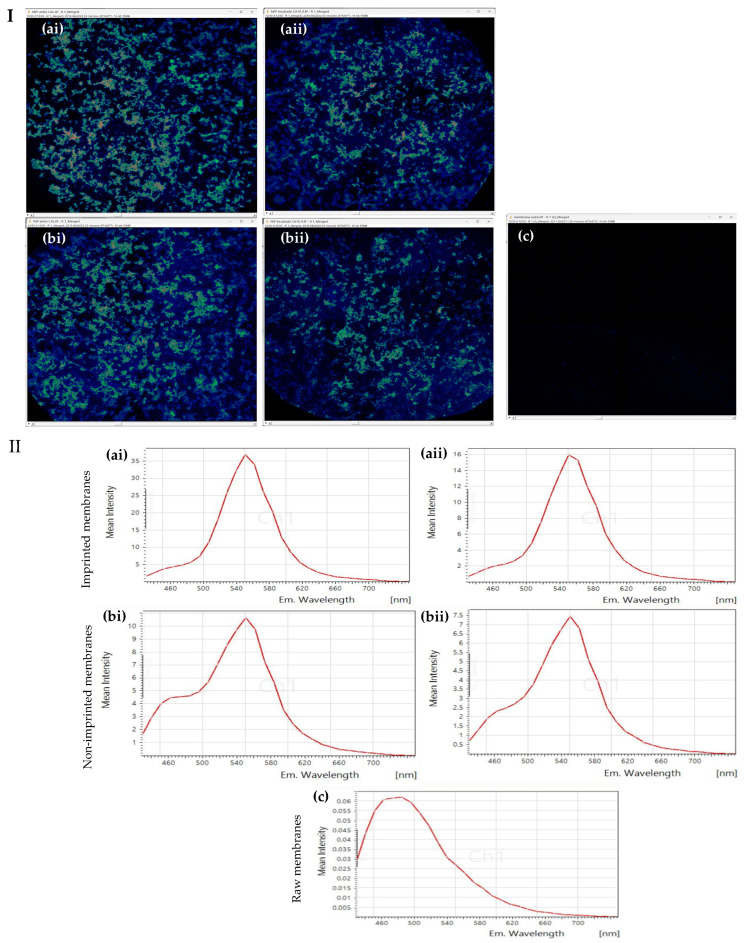
(**I**): Confocal fluorescence images of: imprinted membranes before (**ai**) and after incubation with a CA19-9 standard solution 4.2 kU mL^−1^ in 1% HN in PBS (**aii**), the corresponding non-imprinted controls before (**bi**) and after incubation with the same CA19-9 standard solution (**bii**), and raw polyamide membranes (**c**). (**II**): Corresponding fluorescence spectra evidencing y-QDs and b-CDs emissions (**ai**–**c**). Fluorescence imaging was visualized using the 3-3-2 RGB LUT, with colors reflecting the relative intensities of the acquired fluorescence channels.

The fluorescence behavior of the imprinted membranes was investigated using confocal microscopy and fluorescence spectroscopy, employing dual excitation at 390 nm and 488 nm for selective detection of b-CDs and y-QDs, respectively (Figure 4).

To enhance visualization, all confocal images were processed using a 3-3-2 RGB look-up table (LUT), mapping low, intermediate, and high signal intensities to blue, green, and yellow/red hues, respectively. Under this color scale, b-CDs (emission ≈ 440–480 nm) appear as blue-green tones, while y-QDs (emission ≈ 550 nm) appear in yellow/green hues. Before incubation with CA 19-9, the imprinted membranes exhibited intense fluorescence, around 55 nm, confirming the efficient incorporation of y-QDs within the polymer matrix (Figure 4I(ai),II(ai)). In contrast, the non-imprinted membranes displayed weaker and less uniform fluorescence, suggesting less favorable distribution of the y-QDs within the polymeric matrix (Figure 4I(bi),II(bi)). After incubation with a standard CA 19-9 solution, a pronounced decrease in fluorescence intensity was observed in the imprinted membranes (Figure 4II(aii)). This quenching behavior may be attributed to specific antigen binding within the imprinted cavities, likely inducing conformational rearrangements or Förster Resonance Energy Transfer (FRET) processes that reduce y-QDs emission [39]. The dominance of yellow/green fluorescence in the imprinted membranes prior to incubation may also be enhanced by energy transfer from the template, as after CA 19-9 binding, the emission intensity decreased markedly, consistent with antigen-induced FRET (Figure 4II(aii)) [40,41]. Conversely, non-imprinted membranes showed only slight changes after exposure to CA 19-9, indicating negligible nonspecific interactions (Figure 4II(bii)). Control measurements using raw polyamide membranes confirmed the absence of significant fluorescence, demonstrating that the observed signals originated solely from the incorporated nanomaterials rather than the substrate itself (Figure 4I(c),II(c)).

### 3.5. Analysis of Calibrations

#### 3.5.1. In Dual@MIPs

To establish an internal reference and enable ratiometric fluorescence sensing, MIP@QDs were combined with b-CDs to form colloidal dual@MIPs. Calibrations were performed in 1% serum HN in PBS 10 mM, pH 6.4. HN serum is a commercially available lyophilized reagent intended for routine quality control of inorganic, organic, and enzymatic components, required reconstitution prior to use. To minimize over quenching on the fluorescence signal of both y-QDs and b-CDs, the serum needed to be diluted to 1% in PBS at pH 6.4. Indeed, higher serum concentrations resulted in extensive non-specific quenching of both fluorophores, which precluded reliable ratiometric detection; therefore, sample dilution was essential to maintain the integrity of the fluorescence signal.

**Figure 5:**
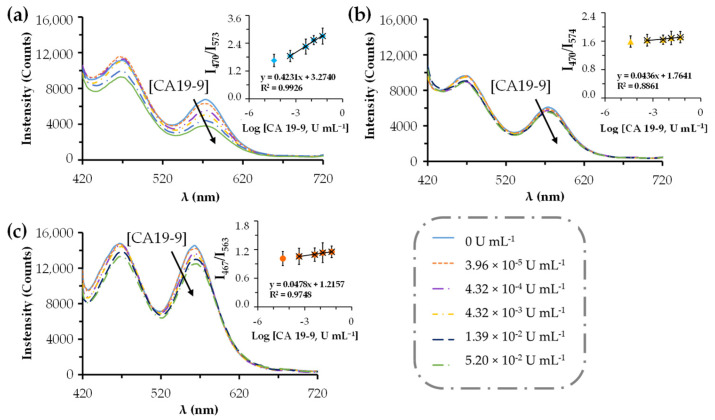
Fluorescence emissions upon calibrations with CA 19-9 standards [3.96 × 10^−5^–5.20 × 10^2^] U mL^−1^ in 1% HN in PBS. Dual@MIPs (**a**); dual@NIPs (**b**); dual@nanoparticles (**c**). MIP@QDs were prepared using CA 19-9 at 1 kU mL^−1^ in PBS 10 mM, pH 6.4. Insets are the corresponding Stern-Volmer plots.

The calibration curves shown in Figure 5 were calculated according to Equation (1) [42]:

(1) $$\frac{I_{0}}{I}=1+k_{sv}\left[Q\right]$$

where *I*_0_ and *I* are the fluorescence intensities in the absence and in the presence of CA19-9 protein, respectively, $k_{sv}$ is the Stern–Volmer constant, and [*Q*] is the concentration of CA19-9 within the interval range tested.

The b-CDs and y-QDs display distinct emission peaks. While b-CDs exhibit an emission peak at 460 nm, the emission maximum of the y-QDs varies slightly between systems, appearing at 573 nm for dual@MIPs, 574 nm for dual@NIPs and 563 nm for the dual@nanoparticles (Figure 5). Upon increasing concentrations of CA 19-9, a decrease in fluorescence intensity at 573 nm is observed for dual@MIPs (Figure 5a). This decrease alters the fluorescence intensity ratio (I_460_/I_573_), which correlates with target concentration.

After the addition of the CA 19-9 standards it was possible to observe a linear decrease in the fluorescence signal of the dual@MIPs from the emission of the y-QDs at 573 nm but not from the b-CDs at 470 nm (Figure 5a). This effect led us to assume b-CDs as a control probe, whereas the y-QDs served as a sensor for the target levels.

The possibility of Förster resonance energy transfer (FRET) between the b-CDs (donor) and the y-QDs (acceptor) was initially considered [39]; however, our experimental results indicate that such interactions are negligible under the conditions employed. The fluorescence intensity of the b-CDs remained essentially constant over the full range of CA 19-9 concentrations (Figure 5), showing no systematic enhancement or reduction associated with y-QD quenching. This stability confirms that the b-CDs are not influenced by the presence of CA 19-9 and therefore function reliably as an internal reference fluorophore for ratiometric detection. Consequently, the observed ratiometric response (I_blue_/I_yellow_) originates exclusively from the selective quenching of the y-QDs upon recognition of CA 19-9 within the imprinted cavities, while the b-CD emission remains target-independent. Any residual energy-transfer effects in this system are more plausibly attributed to proximity interactions between y-QDs and the bound CA 19-9 glycoprotein, rather than between b-CDs and y-QDs, supporting the mechanistic validity of the selected ratiometric design.

The imprinting factor (*IF*) demonstrates de accessibility and mass-transfer resistance of the target through the polymeric matrix, and is evaluated according to the following Equation (2) [42]:

(2) $$IF=k_{sv}(dual@MIPs)/k_{sv}(dual@NIPs)$$

The *IF* reflects how accessible the binding cavities are to the target analyte and how efficiently the antigen diffuses through the polymeric matrix. Higher *IF* values indicate that the imprinted cavities are both more exposed and more easily reached by CA 19-9, whereas lower values suggest greater mass-transfer resistance or limited penetration of the antigen into the polymer network. Thus, IF provides a comparative measure of how effectively molecular imprinting enhances target recognition relative to non-imprinted controls [42]. The fluorescence emissions and the corresponding Stern Volmer plots are shown in Figure 5, and the corresponding data are shown in Table S2.

Indeed, the dual@MIPs showed a $k_{sv}$ of −0.4231, an IF of 9.70 and a LOD of $1.96\times 10^{-5}$ U mL^−1^, with a linear range (LR) of [$4.32\times 10^{-4}$–$5.20\times 10^{-2}$] U mL^−1^ (Table S2). These findings may indicate a controlled rebinding by the recognized process between the target protein and the functional group present in the imprinting cavities, comparatively to its controls dual@NIPs and to dual@nanoparticles.

#### 3.5.2. In Dual@MIPs@mbr

The ratiometric fluorescence calibration of the imprinted membranes and their non-imprinted controls was performed using CA 19-9 standards within the range of 0.4 to 400 U mL^−1^ in 1% human serum (HN) in PBS 10 mM, pH 6.4.

Attempts to perform similar calibrations using dual@nanoparticles directly deposited onto the polyamide membranes were unsuccessful, although recent literature strongly supports the feasibility of embedding QD-based nanomaterials within porous membranes or electrospun fibers to produce stable, luminescent sensing composites [43,44]. The porous architecture of the membrane did not allow adequate retention of the nanoparticles, likely due to weak physical entrapment or insufficient surface interactions. As a result, significant nanoparticle loss occurred during the washing and incubation steps, leading to inconsistent fluorescence signals. Consequently, fluorescence imaging calibration was not performed for these systems. To overcome this limitation and ensure stable nanoprobe immobilization, the dual@nanoparticles were instead incorporated into the membrane via an in situ polymer entrapment strategy. In this optimized procedure, y-QDs were first dispersed in the pre-polymerization mixture, followed by the addition of b-CDs to generate the colloidal dual@MIPs system. This mixture was then assembled onto the polyamide membrane by controlled filtration, forming a thin MIP layer that simultaneously embedded the nanoparticles within the membrane pores. No fluorescence was detected in the filtrate under UV illumination, confirming that the nanoprobes were effectively retained.

**Figure 6:**
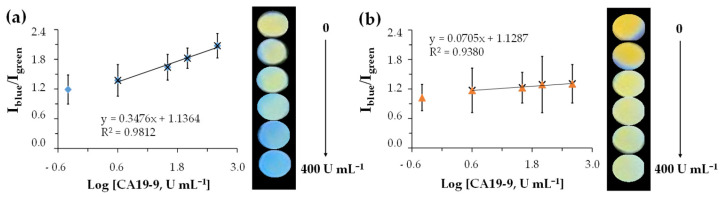
Calibration curves of dual@MIPs@mbr (**a**) and of dual@NIPs@mbr (**b**), in 1% HN in PBS.

The analytical performance of the imprinted membranes was evaluated using fluorescence-based calibration assays with CA 19-9 standards ranging from 0.4 to 400 U mL^−1^ prepared in 1% HN serum in PBS (10 mM, pH 6.4), as shown in Figure 6a. As mentioned earlier in Section 3.5.1., the serum needed to be 1% diluted. Regarding the fluorescence intensity ratio (I_blue_/I_yellow_) of the imprinted membranes showed linearity in the 4–400 U mL^−1^ range, increasing with rising CA 19-9 concentrations. This concentration-dependent trend yielded a linear Stern–Volmer relationship across the studied range, with a $k_{sv}$ of −0.3476, an IF of 4.93 and a LOD of 0.056 U mL^−1^ (Figure S2, Table S3). The non-imprinted membranes showed only small variations in the (I_blue_/I_yellow_) ratio, in the same interval range (Figure 6b). The lowest-concentration data point was excluded from the linear regression because its relatively high variability near the detection limit substantially decreased the R^2^ value and impaired the linearity of the calibration model. Excluding this point allows the calibration to more accurately represent the linear dynamic range of the sensor.

Although the working linear range of dual@MIPs@mbr (4–400 U mL^−1^) appears higher than the normal physiological interval of CA 19-9 (<37 U mL^−1^), this result is from the required 1% serum dilution step, rather than from a limitation of the sensing platform. Because samples are diluted 1:100 to prevent over-quenching in native serum, the CA 19-9 concentration in the original patient specimen is 100-fold higher than the concentration measured during analysis. Thus, a measured value of 4 U mL^−1^ corresponds to 400 U mL^−1^ in undiluted serum, and even the lowest concentration tested, not included in the LR (0.4 U mL^−1^ in diluted form, equivalent to 40 U mL^−1^ in undiluted serum) lies above the clinical cut-off of 37 U mL^−1^. As such, while the method demonstrates adequate analytical resolution for detecting clinically relevant elevations of CA 19-9, the true diagnostic resolution in real patient samples cannot yet be established. Future work will focus on validating the sensor using clinical specimens to determine its resolution, biological variability, and clinical decision-making performance.

A comparative evaluation of the analytical performance of the imprinted membranes with previously reported CA 19-9 sensors is presented in Table 1. The limit of detection obtained in this work (0.056 U mL^−1^) is comparable to or lower than those reported for electrochemical aptasensors, ELISA-type immunoassays, and nanomaterial-based fluorescent biosensors.

Moreover, while most existing systems require sophisticated instrumentation (potentiostats, spectrofluorometers, Raman spectrometers) and rely on fragile biological receptors such as antibodies or aptamers, the proposed imprinted membranes enable instrument-free, visually interpretable detection, use stable synthetic receptors, and exhibit a broader linear range (4–400 U mL^−1^) than many antibody-based formats.

### 3.6. Selectivity of the Imprinted Membranes

**Figure 7:**
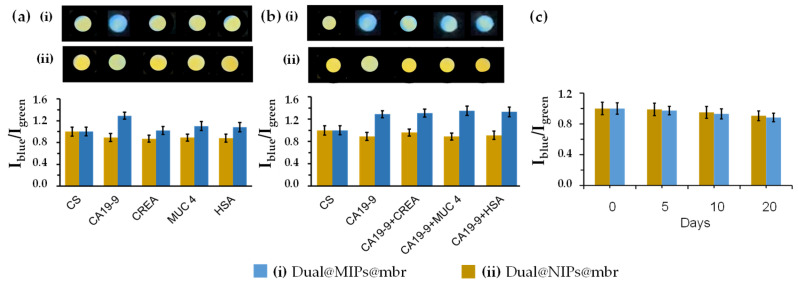
Selectivity of the imprinted membranes (dual@MIPs@mbr) and its controls dual@NIPs@mbr: in the presence of CA 19-9 (40 U mL^−1^) alone (**a**); and in the presence of potential serum interferents: CREA (0.06 µg·mL^−1^), HSA (0.4 mg·mL^−1^), and MUC4 (0.014 ng·mL^−1^) in 1% HN in PBS (RSD%, S/N = 5) (**b**). Images were captured under 365 nm UV light. Stability of the membranes stored dry at 22 °C for 20 days, protected from light (**c**). Green and blue channel intensities (RGB) were measured under 365 nm UV light, and the intensity ratio (I_blue_/I_green_) was calculated from the captured images within RSD limits (S/N = 5).

The selectivity of the imprinted membranes for CA 19-9 was quantitatively evaluated by comparing the blue-to-green fluorescence intensity ratio (I_blue_/I_green_) obtained under identical experimental conditions (Figure 7).

Upon incubation with CA 19-9 at 40 U mL^−1^, the imprinted membranes exhibited a fluorescence ratio (I_blue_/I_green_) enhancement, from 1.00 ± 0.03 (control) to 1.44 ± 0.05, corresponding to an approximately 44% increase relative to the control sample (CS). In contrast, the non-imprinted controls showed a minor variation, from 1.00 ± 0.04 to 1.08 ± 0.06, upon exposure to the same CA 19-9 concentration. The imprinted membranes also maintained strong selectivity in the presence of potential serum interferents. When incubated with creatinine (0.06 µg·mL^−1^), human serum albumin (0.4 mg·mL^−1^), or mucin 4 (0.014 ng·mL^−1^), the (I_blue_/I_green_) ratios remained statistically unchanged at 1.40 ± 0.05, 1.42 ± 0.04, and 1.41 ± 0.06, respectively, values comparable to those obtained for CA 19-9 alone (1.44 ± 0.05). Conversely, non-imprinted membranes showed stable fluorescence ratios across all conditions, 1.07 ± 0.05 (CA 19-9), 1.05 ± 0.05 (CREA), 1.06 ± 0.04 (HSA), and 1.04 ± 0.04 (MUC4), remaining within experimental error of the control (1.00 ± 0.04).

Overall, these quantitative findings demonstrate that the ratiometric sensing mechanism is governed by specific antigen–polymer interactions rather than nonspecific matrix effects, underscoring the strong selectivity and analytical reliability of the imprinted membranes for CA 19-9 detection in complex biological media.

### 3.7. Stability and Reproducibility of the Imprinted Membranes

The stability of the imprinted membranes was quantitatively monitored by measuring the blue-to-green fluorescence intensity ratio (I_blue_/I_green_) over a 20-day period under dry storage at 22 °C, protected from light. As shown in Figure 7c, the imprinted membranes exhibited a modest fluorescence decrease of 8.21% ± 2.12% after 20 days, whereas the non-imprinted controls showed a smaller decrease of 2.67% ± 1.82% under identical conditions (RSD, *n* = 5). These results indicate that the imprinted membranes remain photochemically stable under ambient storage, but only for shorter timeframes, with performance remaining fully reliable for up to 5 days. To ensure long-term applicability in point-of-care formats, further work will investigate vacuum storage, controlled-atmosphere packaging, and moisture-barrier materials as strategies to prevent degradation and enable a ready-to-use clinical product.

Reproducibility was additionally assessed through recovery studies using CA 19-9 standards prepared in 1% human serum (HN) in PBS. Recoveries ranged from 97.71% ± 1.49% at 9.62 × 10^1^ U mL^−1^ to 83.65% ± 1.51% at 3.88 × 10^−1^ U mL^−1^, with a lower recovery of 54.22% ± 1.49% observed at the highest tested concentration (3.81 × 10^2^ U mL^−1^) (Table 2).

The moderate decrease in recovery at the highest analyte concentration likely reflects partial saturation of the imprinted binding sites and potential mass-transfer limitations associated with the large molecular size of the CA 19-9 glycoprotein at elevated concentrations. Because clinically relevant CA 19-9 levels in pancreatitis and pancreatic cancer frequently exceed 100–1000 U mL^−1^, this saturation trend at very high spiked levels does not compromise applicability within clinically significant ranges.

## 4. Conclusions

Dual@MIPs@mbr were successfully developed as a selective and ratiometric fluorescence platform for the detection of the tumor biomarker CA 19-9. Embedding y-QDs and b-CDs within the imprinted polymeric matrix enabled a distinct and quantifiable fluorescence shift from yellow/green to blue in response to increasing CA 19-9 levels. The imprinted membranes exhibited superior analytical performance, with a linear response in the clinically relevant diluted range of 4–400 U mL^−1^ and a low detection limit of 0.056 U mL^−1^. Selectivity assays demonstrated strong discrimination against common serum interferents, and the ratiometric signal (I_blue_/I_green_) was governed by specific antigen–cavity interactions rather than nonspecific fluorescence effects. Short-term stability studies demonstrated photochemical robustness for up to approximately five days, and recovery assays indicated good analytical reproducibility. While promising, the method has not yet been verified using real clinical samples. Thus, the present work establishes the analytical principles and feasibility of the dual@MIPs@mbr approach rather than a fully validated diagnostic tool. Overall, the membranes demonstrate strong potential for integration into low-cost, portable platforms for future point-of-care CA 19-9 assessment, pending further validation using real-world biological samples.

## Figures and Tables

**Table 1:** Comparison of analytical performance of the proposed dual@MIPs@mbr with representative CA 19-9 detection systems reported in the literature.

Detection Method	Recognition Element	LOD (U mL^−1^)	LR (U mL^−1^)	Refs.
Electrochemical	antibody	3 × 10^−6^	1 × 10^−5^–50	[45]
Photoelectrochemical immunosensor	antibody	4 × 10^−4^	1 × 10^−3^–50	[46]
MIP-based fluorescent sensor	MIP	1.58 × 10^−3^	2.76 × 10^−2^–5.23 × 10^2^	[33]
SERS-based immunosensor	antibody	3.43 × 10^−4^	5 × 10^−4^–1 × 10^2^	[47]
MIP-based electrochemical sensor	MIP	3.2 × 10^−3^	5 × 10^−3^–200	[48]
This work: dual@MIPs@mbr	MIP + dual fluorescent nanoprobe	5.6 × 10^−2^	4–400	This work

**Table 2:** Sample analysis and recoveries with the corresponding RSD (%, *n* = 5), Spiked with three concentrations of CA 19-9 protein standards prepared in 1% serum HN in PBS.

Spiked (U mL^−1^)	Found (U mL^−1^)	Recovery ± RSD (%)
$3.88\times 10^{-1}$	$3.25\times 10^{-1}$	83.65±1.51
$9.62\times 10^{1}$	$9.40\times 10^{1}$	97.71±1.49
$3.81\times 10^{2}$	$2.07\times 10^{2}$	54.22±1.49

## Data Availability

The original contributions presented in this study are included in the article/Supplementary Materials. Further inquiries can be directed to the corresponding authors.

## Supplementary Materials

The following supporting information can be downloaded at: https://www.mdpi.com/article/10.3390/s25237363/s1, Figure S1: Fluorescence and absorption spectra recorded during the synthesis and characterization of MIP@QDs and control systems in PBS. (a) Using condition 1 from Table S1; (b) corresponding zoomed region; (c) using the selected condition 2 from Table S1; (d) corresponding zoomed region; (e) fluorescence spectra of dual@MIPs (red solid line), dual@NIPs (yellow solid line) prior to calibration, and b-CDs suspension (blue dot-dash line); (f) UV–Vis absorption spectra of y-QDs (2 mg mL^−1^, green solid line), b-CDs (blue dot-dash line), CA 19-9 standard solution (4.2 kU mL^−1^, brown solid line), and the supernatants collected after template removal from MIP@QDs (purple dotted line) and NIP@QDs (pink dotted line); Figure S2: Calibration of dual@MIPs@mbr (a) and dual@NIPs@mbr (b) using CA 19-9 standards (0.4–400 U mL^−1^) prepared in 1% human serum (PBS 10 mM, pH 6.4, rt). Panels (ai) and (bi) show the variation in blue fluorescence intensity with increasing CA 19-9 concentration; panels (aii) and (bii) show the corresponding variation in green fluorescence intensity; panels (aiii) and (biii) present the ratiometric fluorescence response (I_blue_/I_green_) as a function of the logarithm of CA 19-9 concentration. Analytical parameters for these calibrations are summarized in Table S3; Table S1: Experimental conditions evaluated for the synthesis of MIP@QDs targeting CA 19-9 (1 kU mL^−1^) in PBS; Table S2: Calibration data for dual@nanoparticles, dual@MIPs, and dual@NIPs with CA 19-9 standards in the range [3.96 × 10^−5^ − 5.20 × 10^−2^] U mL^−1^ in 1% HN serum in PBS (10 mM, pH 6.4, rt); Table S3: Calibration data for dual@MIPs@mbr and dual@NIPs@mbr with CA 19-9 standards (0.4–400 U mL^−1^) in 1% HN serum in PBS (10 mM, pH 6.4, rt).
