# Supplementary material for: Molecularly Imprinted Membranes: Dual@MIPs@mbr for On-Site Detection of CA 19-9
Source: Sensors (Basel). 2025 Dec 3;25(23):7363. doi: 10.3390/s25237363 (PMC12694677; doi:10.3390/s25237363)
Supplement: Supplementary file 1 [file sensors-25-07363-s001.zip › sensors-3974429-supplementary.pdf]

Supplementary Materials

Molecularly Imprinted Membranes: Dual@MIPs@mbr for On-Site Detection of CA 19-9

Eduarda Rodrigues [1,†], Ana Xu [1,†], Paula Sampaio [2,3], Rafael C. Castro [4], David S. M. Ribeiro [4], João L. M. Santos [4] and Ana Margarida L. Piloto [1,4,*]

[1] CIETI-LabRISE, ISEP, Polytechnic of Porto, Rua Dr. António Bernardino de Almeida 431, 4249-015 Porto, Portugal; edmfr@isep.ipp.pt (E.R.); naaxu@isep.ipp.pt (A.X.)

[2] i3S—Institute of Research and Innovation in Health, University of Porto, 4200-135 Porto, Portugal; sampaio@i3s.up.pt

[3] IBMC—Institute for Molecular and Cell Biology, University of Porto, 4150-180 Porto, Portugal

[4] LAQV, REQUIMTE, Laboratory of Applied Chemistry, Department of Chemical Sciences, Faculty of Pharmacy, University of Porto, Rua de Jorge Viterbo Ferreira No 228, 4050-313 Porto, Portugal; rafael.castro.cl@hotmail.com (R.C.C.); dsmribeiro@gmail.com (D.S.M.R.); joaolms@ff.up.pt (J.L.M.S.)

* Correspondence: amlpc@isep.ipp.pt

† These authors contributed equally to this work.

Assembly conditions of MIP@QDs and UV-Vis spectra

Table S1 – Experimental conditions evaluated for the synthesis of MIP@QDs targeting CA 19-9 (1 kU mL^{-1}) in PBS.

Condition	QDs (mg)	Polymerization time (min)	Washing time (h)
1	2	30	2
2*	2	30	48

* Condition 2 was selected as the optimized procedure for MIP@QDs synthesis.

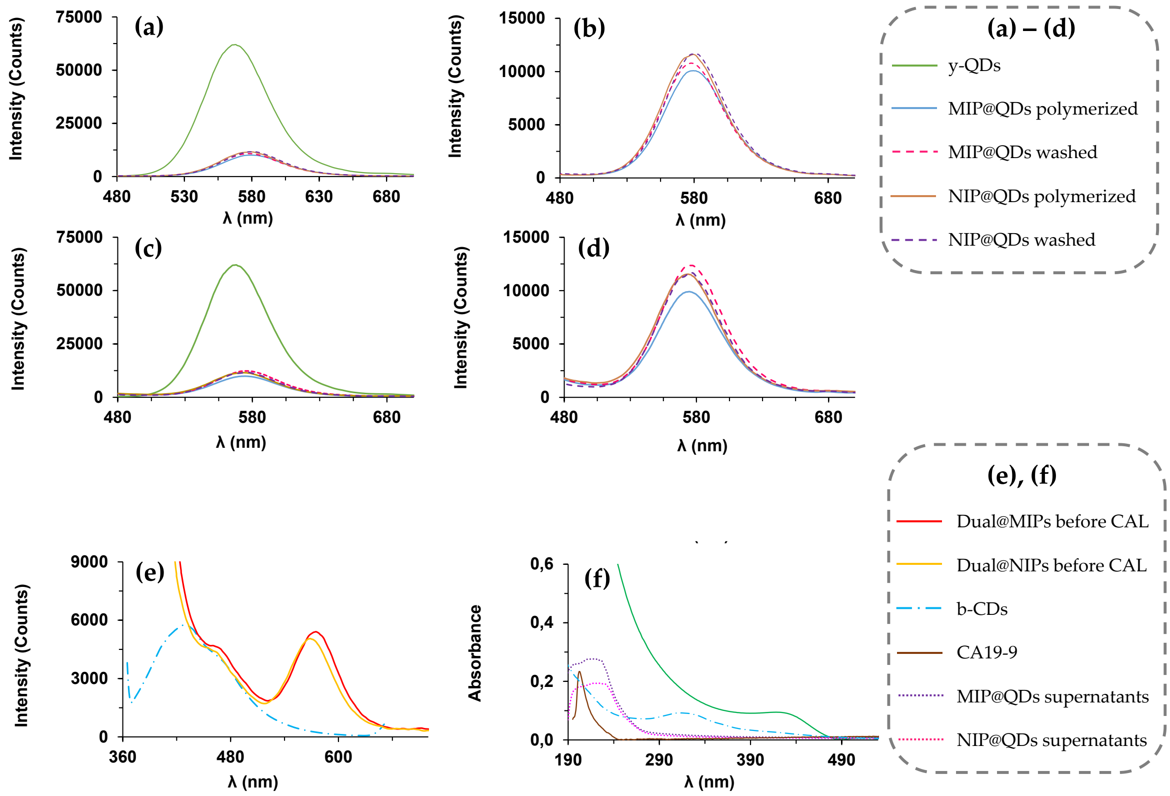

Figure S1. Fluorescence and absorption spectra recorded during the synthesis and characterization of MIP@QDs and control systems in PBS. **(a)** using condition 1 from Table S1; **(b)** corresponding zoomed region. **(c)** using the selected condition 2 from Table S1; **(d)** corresponding zoomed region. **(e)** Fluorescence spectra of dual@MIPs (red solid line), dual@NIPs (yellow solid line) prior to calibration, and of b-CDs suspension (blue dot-dash line). **(f)** UV-Vis absorption spectra of y-QDs (2 mg mL^{-1}, green solid line), b-CDs (blue dot-dash line), CA 19-9 standard solution (4.2 kU mL^{-1}, brown

solid line), and the supernatants collected after template removal from MIP@QDs (purple dotted line) and NIP@QDs (pink dotted line).

Table S2. Calibration data for dual@nanoparticles, dual@MIPs, and dual@NIPs with CA 19-9 standards in the interval range of [$3.96 \times 10^{-5} – 5.20\times 10^{-2}$] U mL^{-1} in 1% HN serum in PBS 10 mM pH 6.4, rt.

Analytical Parameter	dual@nanoparticles	dual@MIPs	dual@NIPs
LR (U mL^{-1})	4.32×10^{-4}-5.20×10^{-2}	4.32×10^{-4}-5.20×10^{-2}	4.32×10^{-4}-5.20×10^{-2}
LOD (U mL^{-1})	5.93×10^{-5}	1.96×10^{-5}	8.91×10^{-5}
k_{sv}	0.0478	0.4231	0.0436
IF		9.70	

Table S3. Calibration data for dual@MIPs@mbr and dual@NIPs@mbr with CA 19-9 standards (0.4 - 400 U mL^{-1}) in 1% HN serum in PBS 10 mM pH 6.4, rt.

Analytical Parameter	dual@MIPs@mbr	dual@NIPs@mbr
LR (U mL^{-1})	4 - 400	4 - 400
LOD (U mL^{-1})	0.056	…
ksv	-0.3476	-0.0705
IF	4.93	…

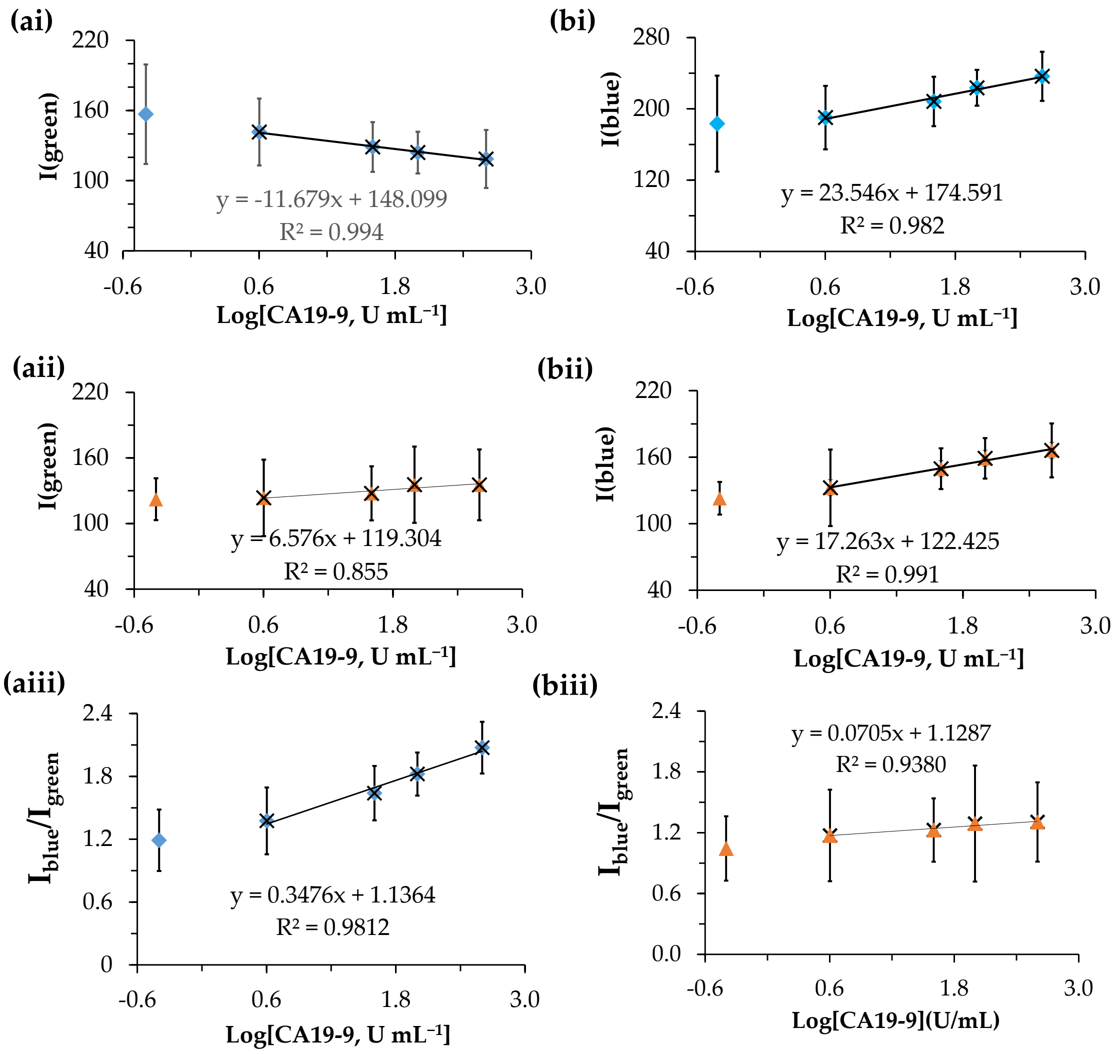

Figure S2. Calibration of dual@MIPs@mbr (**a**) and dual@NIPs@mbr (**b**) using CA 19-9 standards (0.4 - 400 U mL^{-1}) prepared in 1% human serum (PBS 10 mM, pH 6.4, rt). Panels (**ai**) and (**bi**) display the variation in blue fluorescence intensity of the membranes with increasing CA 19-9 concentration, while panels (**aii**) and (**bii**) show the corresponding variation in green fluorescence intensity. Panels (**aiii**) and (**biii**) present the ratiometric fluorescence response (I_{blue}/I_{green}) as a function of the logarithm of CA 19-9 concentration. The analytical parameters derived from these calibrations are summarized in Table S3.
